# Supplementary material for: Role of GDF15/MAPK14 Axis in Chondrocyte Senescence as a Novel Senomorphic Agent in Osteoarthritis
Source: Int J Mol Sci. 2022 Jun 24;23(13):7043. doi: 10.3390/ijms23137043 (PMC9266723; doi:10.3390/ijms23137043)
Supplement: Supplementary file 1 [file ijms-23-07043-s001.zip › ijms-1763348-supplementary.pdf]

SUPPLEMENTARY INFORMATION

Role of GDF15/MAPK14 Axis in Chondrocyte Senescence as a Novel Senomorphic Agent in Osteoarthritis

Pei-Wei Weng [1,2,3,] Narpati Wesa Pikatan [4,5,] Syahru Agung Setiawan [4,6,] Vijesh Kumar Yadav [4,] Iat-Hang Fong [4,] Chia-Hung Hsu [7,8,*] Chi-Tai Yeh [4,9] and Wei-Hwa Lee [4,10,*]

1 Department of Orthopaedics, School of Medicine, College of Medicine, Taipei Medical University,

1. Taipei 11031, Taiwan; wengpw@tmu.edu.tw

2 Department of Orthopaedics, Shuang Ho Hospital, Taipei Medical University, New Taipei City 23561, Taiwan

3 Graduate Institute of Biomedical Materials and Tissue Engineering, College of Biomedical Engineering, Taipei Medical University, Taipei 11031, Taiwan

4 Department of Medical Research & Education, Shuang Ho Hospital, Taipei Medical University, New Taipei City 235, Taiwan; narpatisesa@gmail.com (N.W.P.); vijeshp2@gmail.com (V.K.Y.); impossiblewasnothing@hotmail.com (I.-H.F.); ctyeh@s.tmu.edu.tw (C.-T.Y.); se-tiawan.syahru@gmail.com (S.A.S.)

5 Division of Urology, Department of Surgery, Faculty of Medicine, Universitas Gadjah Mada/Dr. Sardjito Hospital, Yogyakarta 55281, Indonesia

6 International Ph.D. Program in Medicine, College of Medicine, Taipei Medical University, Taipei City 11031, Taiwan

7 Department of Emergency Medicine, Shuang-Ho Hospital, Taipei Medical University, New Taipei City 23561, Taiwan

8 Graduate Institute of Injury Prevention and Control, College of Public Health, Taipei Medical University, Taipei City 11031, Taiwan

9 Department of Medical Laboratory Science and Biotechnology, Yuanpei University of Medical Technology, Hsinchu City 30015, Taiwan

10 Department of Pathology, Shuang Ho Hospital, Taipei Medical University, New Taipei City 23561, Taiwan

*Corresponding author(s):

Chia-Hung Hsu, MD., Ph.D

Department of Emergency Medicine, Shuang-Ho Hospital, Taipei Medical University, New Taipei City, Taiwan. Tel: +886-2-2490088 ext. 8881, Fax: +886-2-2248-0900.

E-mail: 12119@s.tmu.edu.tw
Wei-Hwa Lee, MD., Ph.D
Department of Pathology, Taipei Medical University-Shuang Ho Hospital, New Taipei City, Taiwan. Tel: +886-2-2490088 ext. 8885, Fax:886-3-5401480. E-mail: whlpath97616@s.tmu.edu.tw

Supplementary Table S1. The membranes were incubated in primary antibodies.

No.	Target	Dilution		Source	
1	GDF-15	1:1000	35	NBP2-44214	novusbio
2	p21	1:1000	21	#2947	Cell signaling
3	p-MAPK14	1:1000	43	#4511	Cell signaling
4	MAPK14	1:1000	40	#8690	Cell signaling
5	Bax	1:2000	20	#5023	Cell signaling
6	Bcl-2	1:2000	26	#15071	Cell signaling
7	γ-H2AX	1:5000	15	ab81299	abcam
8	p-ErbB2	1:1000	185	ab53290	abcam
9	ErbB2	1:1000	180	ab134182	abcam
10	p16	1:5000	16	ab51243	abcam
11	β-Gal	1:2000	76	sc-377257	Santa Cruz
12	GAPDH	1:10000	37	60004-1-Ig	PROTEINTECH

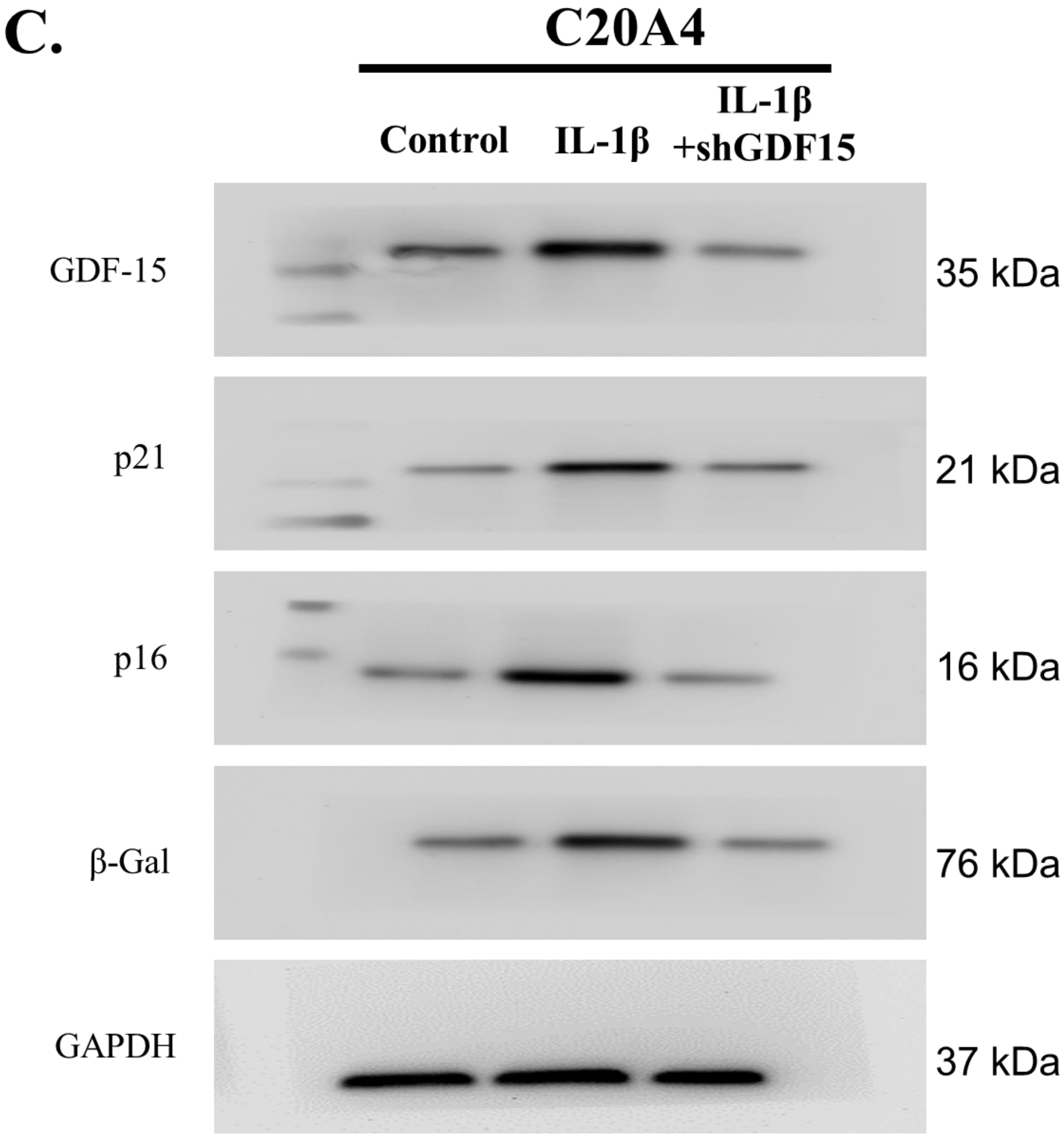

Supplementary Figure S1. Full-size blots of Figure 3C

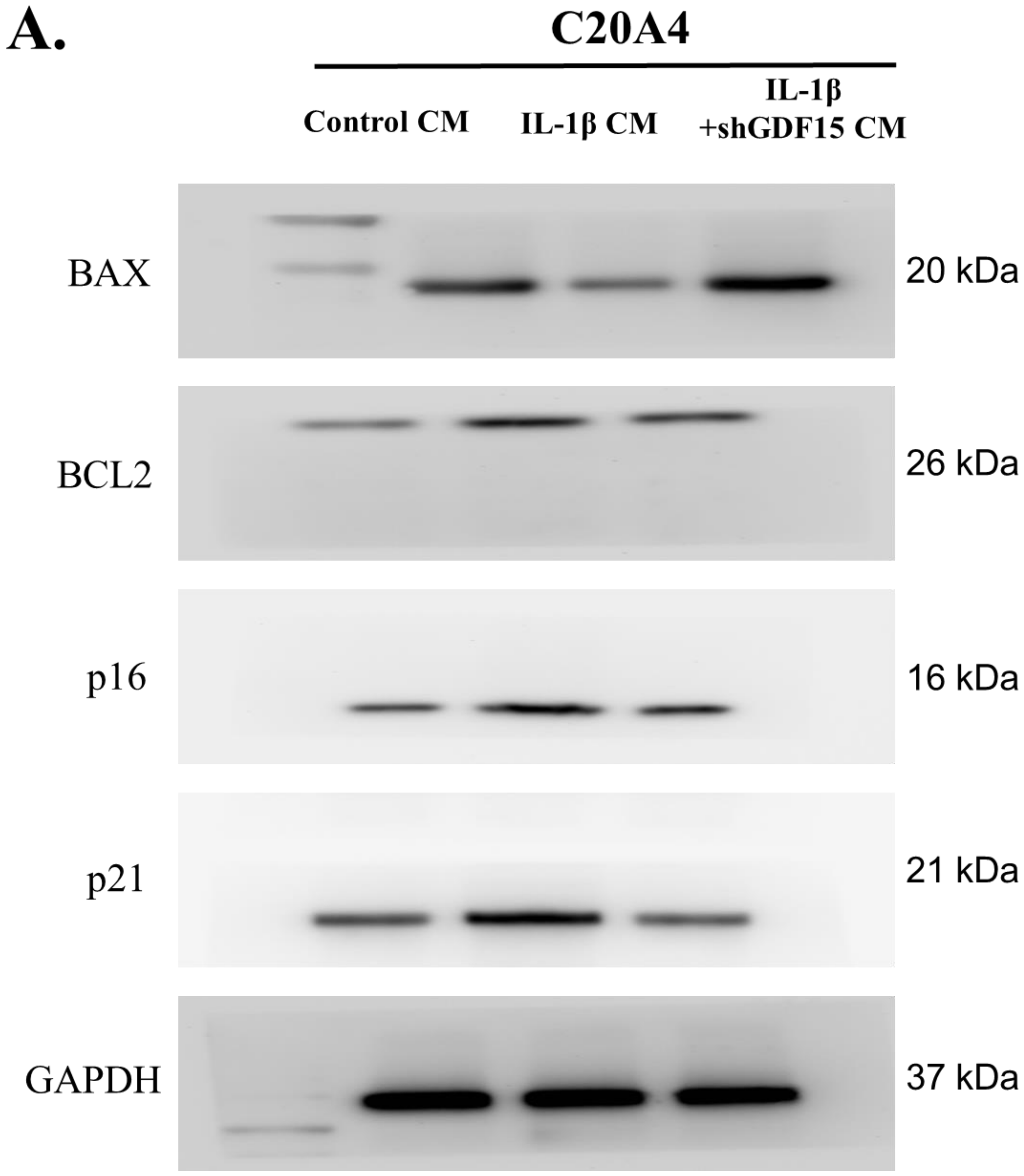

Supplementary Figure S2. Full-size blots of Figure 4A

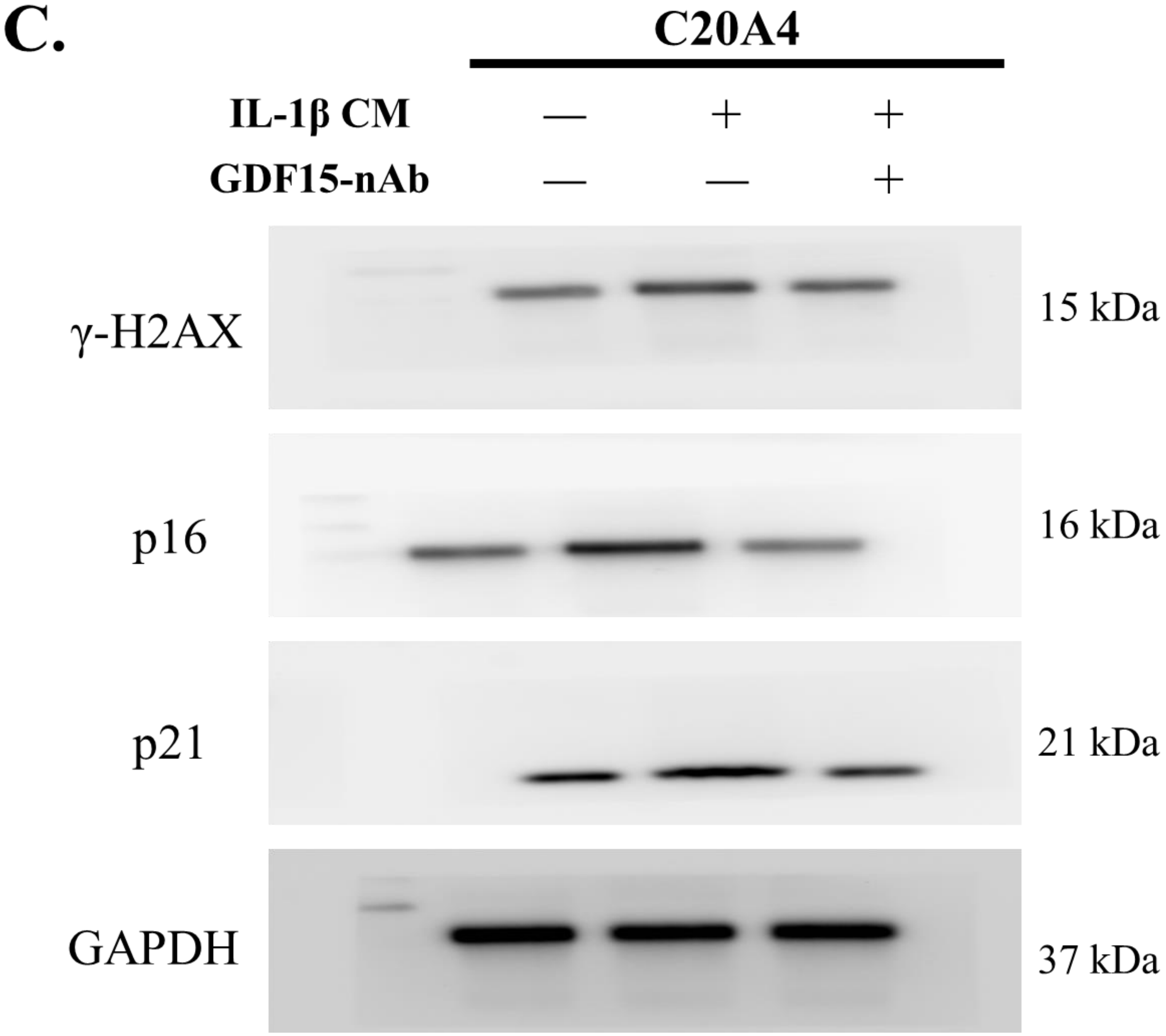

Supplementary Figure S3. Full-size blots of Figure 5C

A.

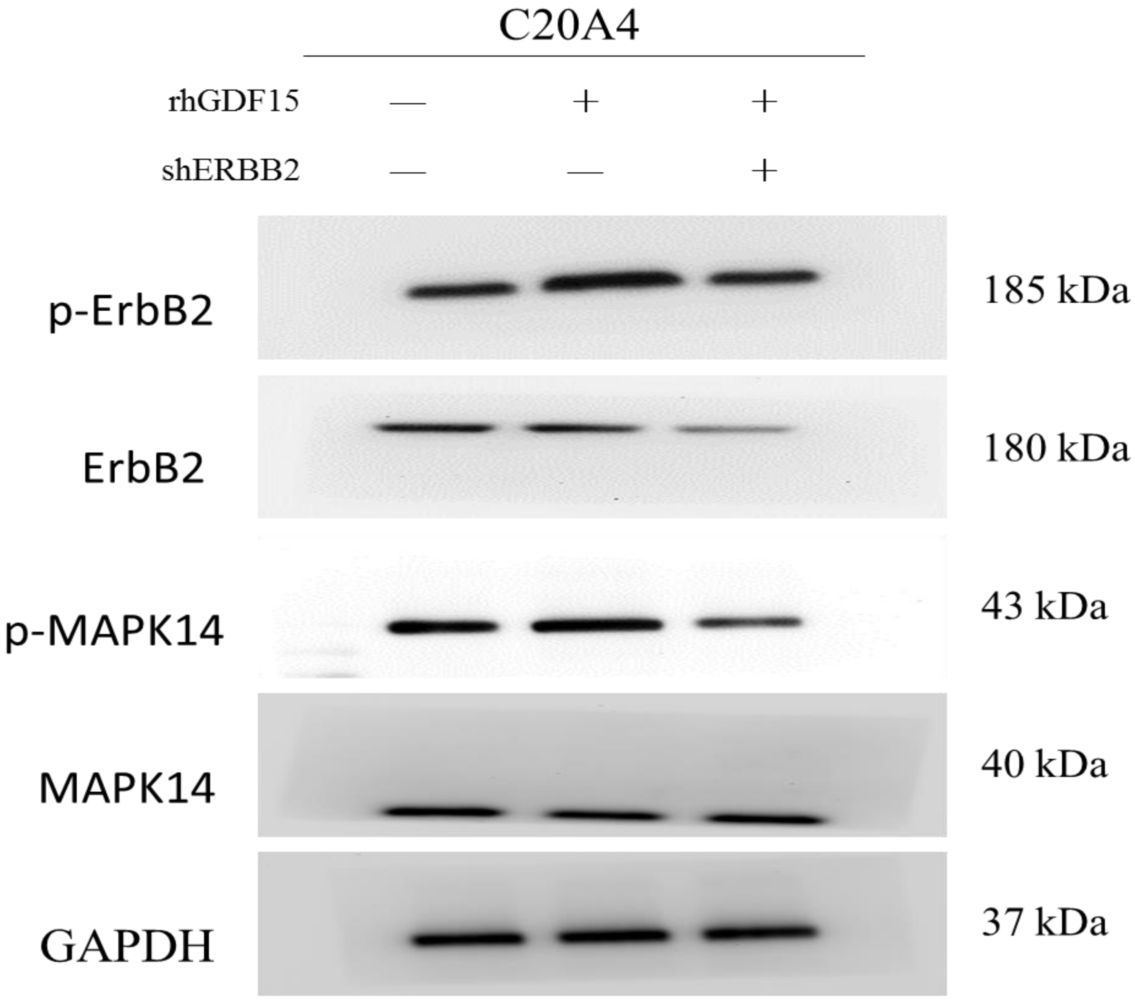

B.

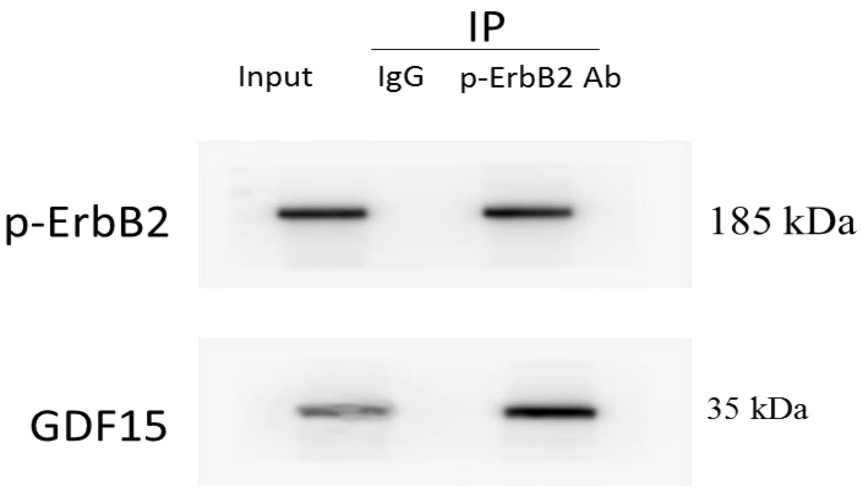

Supplementary Figure S4. Full-size blots of Figure 6A and 6B
